# Autonomous Magnetic Navigation in Endoscopic Image Mosaics

**DOI:** 10.1002/advs.202400980

**Published:** 2024-03-14

**Authors:** Michelle Mattille, Quentin Boehler, Jonas Lussi, Nicole Ochsenbein, Ueli Moehrlen, Bradley J. Nelson

**Affiliations:** ^1^ Multi‐Scale Robotics Lab ETH Zurich Tannenstrasse 3 Zurich 8092 Switzerland; ^2^ Department of Obstetrics University Hospital of Zurich Rämistrasse 100 Zürich 8092 Switzerland; ^3^ The Zurich Center for Fetal Diagnosis and Therapy University of Zurich Rämistrasse 71 Zürich 8092 Switzerland; ^4^ Department of Pediatric Surgery University Children's Hospital Zurich Steinwiesstrasse 75 Zürich 8092 Switzerland

**Keywords:** autonomous navigation, fetal surgery, magnetic navigation, medical robotics, mosaicking

## Abstract

Endoscopes navigate within the human body to observe anatomical structures with minimal invasiveness. A major shortcoming of their use is their narrow field‐of‐view during navigation in large, hollow anatomical regions. Mosaics of endoscopic images can provide surgeons with a map of the tool's environment. This would facilitate procedures, improve their efficiency, and potentially generate better patient outcomes. The emergence of magnetically steered endoscopes opens the way to safer procedures and creates an opportunity to provide robotic assistance both in the generation of the mosaic map and in navigation within this map. This paper proposes methods to autonomously navigate magnetic endoscopes to 1) generate endoscopic image mosaics and 2) use these mosaics as user interfaces to navigate throughout the explored area. These are the first strategies, which allow autonomous magnetic navigation in large, hollow organs during minimally invasive surgeries. The feasibility of these methods is demonstrated experimentally both in vitro and ex vivo in the context of the treatment of twin‐to‐twin transfusion syndrome. This minimally invasive procedure is performed in utero and necessitates coagulating shared vessels of twin fetuses on the placenta. A mosaic of the vasculature in combination with autonomous navigation has the potential to significantly facilitate this challenging surgery.

## Graphical abstract

This paper proposes the first automated navigation strategies to explore and navigate in large, hollow anatomical structures with remote magnetic navigation during minimally invasive procedures. The methods relay on endoscopic image mosaics to provide surgeons with a map of the environment for surgical planning and localization. This approach promises to facilitate such procedures for the surgeons and enhance patient safety.

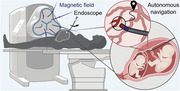

## 1 Introduction

Remote magnetic navigation (RMN) is an emerging field with high potential for surgical robotics.^[^ ^1^ ^]^ Magnetic guidewires, catheters, or endoscopes are steered inside the human body with magnetic fields generated by an electromagnetic navigation system (eMNS) placed next to the patient.^[^ ^2^, ^3^, ^4^ ^]^

By remotely changing the orientation of the magnetic fields, the magnetic material inside the surgical tools experiences a magnetic torque to align with the magnetic field orientation resulting in bending the distal part of the flexible tools.^[^ ^5^ ^]^ In this way, the instrument tips are controlled in a precise and highly dexterous manner. A limitation of soft, flexible instruments is their inability of generating large interaction forces in a stable way with the surrounding anatomy. This property naturally leads to increased procedural safety and makes RMN an excellent candidate for endoscopic procedures, where tissue interactions are to be minimized while the instrument is navigated in unknown anatomical environments.^[^ ^6^, ^7^, ^8^ ^]^ In 2003, Stereotaxis Inc. introduced RMN into the clinics for cardiac ablations.^[^ ^9^ ^]^ This led to an increased maneuverability of the catheters compared to manual guidance and facilitated steering into anatomically challenging areas. Their procedures have also been reported to be safe and efficacious, and to reduce operator fatigue.^[^ ^3^ ^]^ RMN has also been proposed for a variety of natural orifice and minimally invasive procedures such as colonoscopies, endovascular procedures, neurosurgery, bronchoscopy, and fetal surgery.^[^ ^2^, ^4^, ^6^, ^8^, ^10^, ^11^, ^12^, ^13^, ^14^ ^]^

One of the major shortcomings of minimally invasive surgeries in large, hollow organs is the narrow field of view of the endoscopes. Surgeons must memorize the tool's environment for surgical planning, navigation, localization, and to characterize areas of interest.^[^ ^15^, ^16^, ^17^, ^18^, ^19^ ^]^ To address this issue, several groups have proposed generating mosaics or 3D reconstructions from endoscopic images. 3D reconstructions of the bladder and the stomach have been generated with recordings from cystoscopies and gastroscopies to facilitate cancer detection and surveillance,^[^ ^15^, ^16^, ^17^ ^]^ Mosaicking of endoscopic images was proposed for generating maps of the vascular anatomy of the placenta for the treatment of twin‐to‐twin transfusion syndrome (TTTS).^[^ ^18^, ^20^, ^21^, ^22^ ^]^ TTTS is a severe complication of monochorionic twin pregnancies and is characterized by a unbalanced blood flow from one twin to the other over shared vessels on the placenta (anastomoses), which leads to a severe amniotic fluid discrepancy between the twins.^[^ ^23^ ^]^ The gold standard treatment is a minimally invasive procedure called fetoscopic laser coagulation (FLC). The surgeon first identifies all anastomoses by navigating the endoscope over the placenta while memorizing the vascular anatomy. Next, the endoscope is navigated to all anastomoses to coagulate them with a laser.^[^ ^24^, ^25^ ^]^ Endoscopic image mosaics have the potential to significantly facilitate the identification of anastomoses, because the field of view of the endoscope is too small to see the required placental vasculature in a single endoscopic image.^[^ ^18^, ^20^, ^21^, ^22^ ^]^

Mosaics of endomicroscopic images have been proposed for the enhancement of probe‐based confocal laser endomicroscopy (pCLE).^[^ ^19^, ^26^, ^27^ ^]^ pCLE is an imaging modality to generate high resolution microscopic tissue images for optical biopsy.^[^ ^19^, ^27^ ^]^ Mosaicking during pCLE requires a slow and controlled scanning motion with sub‐millimeter accuracy while maintaining optimal tissue contact.^[^ ^27^ ^]^ The automation of this scanning motion was proposed for robotic surgical systems such as the da Vinci robot.^[^ ^19^, ^26^, ^27^ ^]^ Trajectories were planned in the camera frame or a frame fixed with respect to the base of the robot respectively and steered the probe along the trajectories with visual servoing.^[^ ^19^, ^26^, ^27^ ^]^

Autonomous navigation of surgical instruments promises to facilitate procedures and enhance patient safety.^[^ ^28^ ^]^ It has the potential to increase precision and reduce the impact of human factors, such as stress or fatigue, and eliminate the influence of tremors in the hands.^[^ ^1^, ^29^ ^]^ Less experienced surgeons would be able to focus more on critical procedure‐related tasks than on tool navigation, and with fewer tasks to learn, the required training time to master a procedure would be significantly reduced.^[^ ^28^, ^29^ ^]^ Despite these advantages, no commercial solution for autonomous navigation of surgical tools is available yet for RMN. In research, promising preliminary results for automated steering with RMN were shown for bronchoscopy,^[^ ^4^ ^]^ endovascular procedures,^[^ ^10^, ^30^ ^]^ neurosurgery,^[^ ^12^ ^]^ and fetal surgery.^[^ ^14^ ^]^ Martin et al. demonstrated autonomous navigation during an in vivo colonoscopy on a porcine model.^[^ ^8^ ^]^ No autonomous navigation with RMN in large, hollow anatomical structures such as the uterus, stomach, or bladder has been performed so far.

Our work was motivated by FLC to treat TTTS. As shown in our previous work, RMN has great potential for FLC.^[^ ^14^ ^]^ Substituting the current rigid endoscopes with flexible, steerable models enables access to the entire placenta for all placental locations, while enhanced angles between the endoscope and the placenta during the vessel coagulation mitigate the risk of injuring surrounding tissue.^[^ ^14^ ^]^ In this previous work, we demonstrated the potential of robot‐assisted navigation for FLC with automated adjustment motions to reach targets in the current field of view of the endoscope. However, regions of interest and the majority of the targets often lie outside of this limited area. To reach these locations, the vascular anatomy still had to be memorized, and the endoscope was controlled with a manual controller. These constitute major limitations, which are addressed in the present work, by proposing the first autonomous navigation strategies for large, hollow organs with magnetic actuation. These allow to reach locations beyond the limited field of view of the endoscopic camera during minimally invasive surgeries. The proposed methods can handle various visual conditions including challenging low‐texture environments, which are typical in surgical procedures. We propose two automated, model‐free control strategies for RMN relying on endoscopic mosaics. The main contributions are: 1) a visual servoing navigation strategy to explore the environment autonomously from a given starting position while generating an endoscopic mosaic, 2) a navigation method to reach any target in the generated mosaic, 3) a quantitative evaluation of the proposed navigation strategies in simplified in vitro settings, and 4) the demonstration of the proposed methods for FLC by navigating over a human ex vivo placenta underwater. In this context, this has the potential to significantly facilitate the surgery as it automates the navigation of the endoscope during the procedure. It provides to the surgeons with a map of the placental vascular anatomy, where target locations can easily be identified and reached.

In Section 2, the proposed navigation strategies and magnetic actuation are described in detail and the results of the evaluation in vitro and ex vivo are presented and discussed. The conclusions are drawn and an outlook is provided in Section 3. The experimental setup is explained in Section 4.

## 2 Results and Discussion

### 2.1 Working Principle

**Figure 1:**
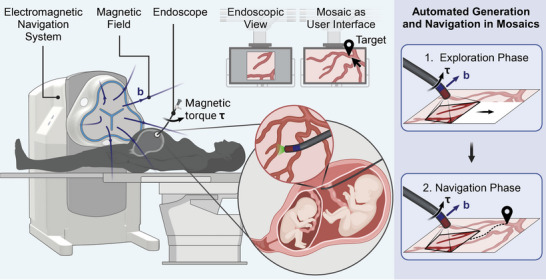
Overview of the remote magnetic navigation platform for automated endoscopic navigation for twin‐to‐twin transfusion syndrome treatment. The electromagnetic navigation system is positioned next to the patient and generates a magnetic field b. The magnetic fields create a magnetic torque τ on a magnetic endoscope to control the orientation of its tip. We propose two automated navigation methods relying on mosaics: 1) autonomous exploration of the environment while the endoscopic images are stitched into a mosaic and 2) use of the mosaic as a graphical user interface where target positions can be selected to which the endoscope is automatically guided.

**Figure** **1** shows an overview of our platform and how it can be used for fetal surgery. The eMNS is placed next to the patient and generates magnetic fields to steer the magnetic endoscope. The procedure consists of two phases depicted on the right‐hand side of Figure 1:

1. Exploration phase, where the endoscope is controlled to autonomously explore the environment while the endoscopic images are stitched into a mosaic in real‐time. This provides the surgeons with a map of the vascular anatomy instead of having to memorize it (Section 2.3.2).
2. Navigation phase, where the mosaic generated in Phase 1 is used as an interactive map. The user can select target locations to which the endoscope will be automatically guided (Section 2.3.3).

### 2.2 Magnetic Actuation

In this work, we navigate a flexible endoscope with RMN. The endoscope has permanent magnets embedded in its distal part that bends when exposed to an external magnetic field b due to the magnetic torque τ acting on each magnet. The magnetic torque τ is computed as

(1) τ=m×b

where m is the dipole moment of the magnet.^[^ ^5^ ^]^ The magnetic field is generated by the Navion eMNS, a pre‐clinical system consisting of three current‐controlled electromagnets (see Figure 1). Navion has already demonstrated its ability to navigate a variety of magnetic continuum robots for medical applications.^[^ ^11^, ^13^, ^14^ ^]^ The magnetic field b at any position $p\in R^{3}$ generated by the system is modeled as a linear mapping between b(p) and the electrical currents flowing through the electromagnets $i\in R^{3}$:^[^ ^5^ ^]^

(2) $$i=A^{†}\left(p\right)b\left(p\right)$$

where $A^{†}$ is the Moore‐Penrose pseudoinverse of the actuation matrix A, which is obtained through calibrating the eMNS.^[^ ^31^ ^]^

### 2.3 Navigation

#### 2.3.1 Closed‐Loop Visual Servoing

**Figure 2:**
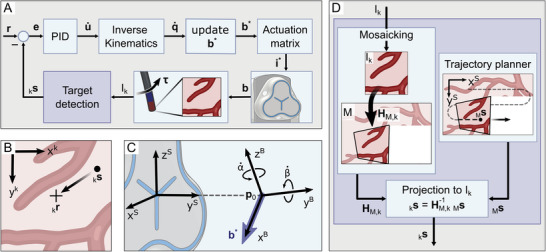
Closed‐loop visual control method. A) Control diagram to move a target 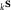 to a reference position 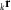. B) Shows the aim of the control in the endoscopic image frame “k”, in which the control is performed. C) The magnetic field frame “B” is rotated incrementally using the intrinsic Euler angles $\dot{\alpha }$ and $\dot{\beta }$. The desired magnetic field vector $b^{*}$ with a magnetic field strength b is attached to the magnetic field frame such that 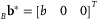.

During both the exploration and navigation phases, the endoscope is controlled in a closed‐loop mode using an extended version of the hand‐in‐eye visual servoing approach proposed in our previous work.^[^ ^14^ ^]^ The control diagram is illustrated in **Figure** **2**A.

The aim is to control the magnetic field to generate the motion of a feature in the current endoscopic image $I_{k}$. The induced magnetic torque (Equation (1)) will cause the endoscope's tip to bend such that a target $s\in Z^{2}$ with pixel coordinates 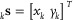 in the image frame (subscribed “k”) moves toward the reference coordinates 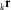 (see Figure 2B). The method to detect 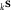 in the endoscopic images depends on the navigation strategy and is described in Sections 2.3.2 and 2.3.3. In both cases, 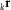 is the center of $I_{k}$.

To ensure an exponential decay of the error 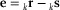 between the reference and the target position, a proportional–integral–derivative (PID) controller is used. It is defined as

(3) $$\dot{u}=\left(K_{p}e+K_{i}\int edt+K_{d}\dot{e}\right)$$

where $\dot{u}$ is the control signal and $K_{p}$, $K_{i}$, and $K_{d}$ are the control parameters, which are found through empirical tuning looking for a stable behavior and minimal overshoots.

The desired magnetic field $b^{*}$ is generated by the eMNS at the position $p_{0}$ in space (see Figure 2C). The field is calculated by intrinsically rotating the desired magnetic field vector from the previous iteration around the axis of a rotating magnetic field frame (denoted “B” in Figure 2C). The “B” frame is attached to the desired magnetic field vector $b^{*}$ such that the coordinates of the desired magnetic field are always expressed as 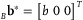, where b is the magnetic field magnitude. At each iteration, “B” is rotated first around its *y* ^*B*^ axis with $\dot{\beta }$ and then around its *z* ^*B*^ axis with $\dot{\alpha }$. The intrinsic Euler angle velocities $\dot{q}={\left[\begin{array}{cc} \dot{\alpha } & \dot{\beta } \end{array}\right]}^{T}$ are obtained from the inverse kinematics. The coordinates of 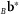 are then transformed from the “B” frame into the magnetic system frame (denoted “S” in Figure 2C), which is attached to the eMNS and in which the system was calibrated.

The kinematics of the image motion are modeled by the Jacobian matrix $J\in R^{2\times 2}$, which maps the magnetic field rotation velocity vector $\dot{q}={\left[\begin{array}{cc} \dot{\alpha } & \dot{\beta } \end{array}\right]}^{T}$ to the motion of the image feature 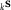 as
(4)

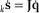




The matrix J is determined through a calibration procedure performed prior to the navigation. The procedure consists of generating two orthogonal exploration motions $\Delta q_{1}$ and $\Delta q_{2}$, and measuring the corresponding displacements in the image $\Delta s_{1}$ and $\Delta s_{2}$. The estimated Image Jacobian $\hat{J}$ is then computed as

(5) $$\hat{J}=\left[\begin{array}{cc} \Delta s_{1} & \Delta s_{2} \end{array}\right]{\left[\begin{array}{cc} \Delta q_{1} & \Delta q_{2} \end{array}\right]}^{-1}$$

Assuming $\hat{J}$ has full rank and a constant 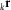, the rotation velocity of the magnetic field vector is computed as

(6) $$\dot{q}={\hat{J}}^{-1}\dot{u}$$

Using Equation 2, the desired input electric currents $i^{*}$ for the eMNS are found to generate $b^{*}$.

#### 2.3.2 Phase 1: Exploration Phase

The endoscope is steered autonomously to explore its environment while stitching its images into a mosaic. The mosaicking is described in detail in Section 2.3.2 and the exploration motions in Section 2.3.2.

##### Mosaicking

During the exploration phase, endoscopic images are stitched into a mosaic using a method adapted from Alabi et al. whose algorithm was validated on in human surgical videos.^[^ ^18^ ^]^ The aim is to find the homography $H_{M,k}$ to project the current endoscopic image $I_{k}$ onto the mosaic. Instead of projecting the images with the affine transformations as in the original paper, we chose to use homographies. Affine transformations were chosen because they yield better results in distorted images.^[^ ^32^ ^]^ Unlike the cameras from the surgical videos used by Alabi et al., our camera is calibrated, and, therefore, the distortion in our images can be corrected.

The method for mosaicking is summarized as follows: when an image is received, its distortion is corrected and the image is cropped. Next, the pixels between $I_{k}$ and the previous endoscopic image $I_{k-1}$ are matched by estimating the optical flow between the images with the neural network FlowNet‐2.

If ${\left[\begin{array}{cc} x_{k-1} & y_{k-1} \end{array}\right]}^{T}$ are the coordinates of each pixel in $I_{k-1}$ and ${\left[\begin{array}{cc} u_{k-1} & v_{k-1} \end{array}\right]}^{T}$ denote the corresponding optical flow, the estimated coordinates of the pixels in $I_{k}$ are

(7) $$\left[\begin{array}{c} x_{k}\\ y_{k} \end{array}\right]=\left[\begin{array}{c} x_{k-1}\\ y_{k-1} \end{array}\right]+\left[\begin{array}{c} u_{k-1}\\ v_{k-1} \end{array}\right]$$

The homography $H_{k-1,k}\in R^{3\times 3}$ to project $I_{k}$ onto $I_{k-1}$ is estimated from the matched pixels using the Random Sample Consensus (RANSAC) method. $H_{k-1,k}$ is then refined using only the inliers through Levenberg–Marquardt optimization. The projection of $I_{k}$ onto $I_{k-1}$ is defined as

(8) $$\lambda \left[\begin{array}{c} x_{k-1}\\ y_{k-1}\\ 1 \end{array}\right]=H_{k-1,k}\left[\begin{array}{c} x_{k}\\ y_{k}\\ 1 \end{array}\right]$$

The perspective transformation $H_{M,k}$ to warp the $I_{k}$ onto the mosaic frame is then found by left‐hand matrix multiplication of all pairwise homographies as

(9) $$H_{M,k}=H_{M,0}\prod_{i=1}^{k}H_{i-1,i}$$

where $H_{M,0}$ is the projection of the first image $I_{0}$ onto the mosaic. $H_{M,0}$ is usually a translation of the image to the center of the mosaic. The image $I_{k}$ is then warped with $H_{M,k}$ and overlaid onto the mosaic.

##### Exploration Motions

To explore the environment autonomously from any given starting position, the endoscope is controlled with the visual servoing method described in Section 2.3.1. Figure 2D illustrates how the target 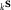 is found for each image $I_{k}$. The aim is that the centers of the projected images in the mosaic follow a predefined trajectory (e.g., a raster as illustrated in Figure 2D) in the mosaic frame. To ensure that the generated mosaic does not contain holes, the trajectory is defined in the mosaic frame and the spacing between neighboring regions is chosen such that the images overlap to account for warping of the images. The target 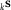 in the current image is found by projecting a target 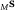 from the mosaic frame (denoted “M”) to the current image (see Figure 2). If 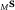 is the point on the trajectory in the mosaic frame, s is obtained with

(10)

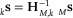

where $H_{M,k}^{-1}$ denotes the inverse of the homography $H_{M,k}$, which projects the current endoscopic image $I_{k}$ on the mosaic and is continuously obtained during the mosaicking (see Section 2.3.2).

#### 2.3.3 Phase 2: Navigation in the Mosaic

**Figure 3:**
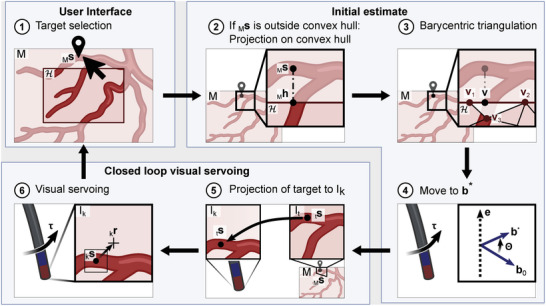
Workflow of the navigation in the mosaic if the target is outside the convex hull H calculated from the projected image centers of the endoscopic images forming the mosaic. The target location 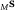 is selected in the mosaic. If it is outside of H, it is projected onto the closest point h on H. Over barycentric interpolation over the magnetic fields at the projected image centers, the desired magnetic field $b^{*}$ at t, the target location, is estimated. The magnetic field vector is then incrementally rotated from the initial magnetic field orientation $b_{0}$ to $b^{*}$ around the axes e, which is perpendicular to the plane spanned by the two vectors. Changing the magnetic field orientation induces a different magnetic torque τ on the endoscopes tip, causing it to bend and controlling its orientation. Next, 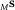 is projected onto the current endoscopic image and is tracked over the following images while the endoscope is steered with visual servoing to bring it to the endoscopic image center.

The mosaic is used as a user interface where surgeons can select target locations by clicking on the desired position in the image (see **Figure** **3**). We assume a magnetic field $b_{0}$ at this point in time. The user selects a target position 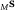 in the mosaic frame by clicking on the mosaic (see Figure 3.1). Once the target location is selected, the endoscope is automatically steered to the selected location in two steps using magnetic navigation:

1. Initial estimate: The magnetic field at the target location is estimated through a barycentric interpolation over the explored locations.
2. Closed‐loop visual servoing: Once the endoscope is moved to the estimated location, the control switches to closed‐loop visual servoing to reach the target more accurately.

**Step 1**: Initial estimate through barycentric interpolation: The aim is to find the magnetic field vector $b^{*}$ at which the target feature 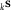 is at the center of the endoscopic image. The vector is estimated through barycentric interpolation over the projected image centers of the images forming the mosaic and their corresponding magnetic field vectors. We define the convex hull H as the smallest convex shape containing the projected image centers. If 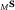 lies outside of the convex hull H, $b^{*}$ would have to be extrapolated. To avoid this, 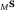 is projected onto the closest point 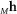 on H and the magnetic field is estimated at this position (see Figure 3.2).

Let v be the position in the mosaic for which the magnetic field will be estimated. If the target 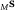 is located outside of H, v corresponds to 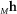 and if 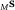 is located inside of H, v is equal to 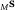. To find the three image centers forming the vertices $v_{i}={\left[\begin{array}{cc} x_{i} & y_{i} \end{array}\right]}^{T}$ of the triangle surrounding v the Delaunay triangulation over all projected image centers is calculated (see Figure 3.3). The vertices represented by v are then converted from cartesian ${\left[\begin{array}{cc} x & y \end{array}\right]}^{T}$ to baricentric coordinates ${\left[\begin{array}{ccc} \lambda_{1} & \lambda_{2} & \lambda_{3} \end{array}\right]}^{T}$ with:

(11) $$\begin{array}{cc} \lambda_{1} & =\frac{\left(y_{2}-y_{3}\right)\left(x-x_{3}\right)+\left(x_{3}-x_{2}\right)\left(y-y_{3}\right)}{d} \end{array}$$

(12) $$\begin{array}{cc} \lambda_{2} & =\frac{\left(y_{3}-y_{1}\right)\left(x-x_{3}\right)+\left(x_{1}-x_{3}\right)\left(y-y_{3}\right)}{d} \end{array}$$

(13) $$\begin{array}{cc} \lambda_{3} & =1-\lambda_{1}-\lambda_{2} \end{array}$$

where $d=(y_{2}-y_{3})(x_{1}-x_{3})+(x_{3}-x_{2})(y_{1}-y_{3})$.

The magnetic field vectors are expressed in spherical coordinates $b^{*}={\left[\begin{array}{ccc} b_{r} & b_{\theta } & b_{\varphi } \end{array}\right]}^{T}$, as then only $b_{\theta }\left(v\right)$ and $b_{\varphi }\left(v\right)$ have to be calculated and $b_{r}$ remains constant. The magnetic field vector components $b_{i}\left(v\right)$ are then interpolated with

(14) $$b_{i}\left(v\right)=\sum_{i=1}^{3}\lambda_{i}b_{i}\left(v_{i}\right)$$

The endoscope is steered from $b_{0}$ to $b^{*}$ by incrementally rotating the magnetic field vector around the axis e perpendicular to the plane spanned by $b_{0}$ and b with a constant angular speed until $b^{*}$ is reached (see Figure 3.4).

**Step 2**: Closed‐loop visual servoing: In this second step, we assume that the selected target is visible in the current endoscopic image $I_{k}$. The closed‐loop visual servoing method proposed in our previous work^[^ ^14^ ^]^ is used to reach the target in the endoscopic image more accurately. The visual servoing method requires the target 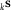 in $I_{k}$. Given the target 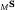 selected in the mosaic, we first identify the image $I_{t}$ from which the pixel of the target in the mosaic was projected. The target 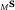 can be projected into this image frame using the inverse of the homography $H_{M,t}$, which was used to project $I_{t}$ on the mosaic during the exploration phase

(15)

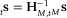

Next, the homography $H_{k,t}$ to project $I_{t}$ onto $I_{k}$ is found using the LoFTR‐matcher method presented in Ref. [33]. It was shown to be robust for stitching in human endoscopic images from FLC.^[^ ^33^ ^]^

The target 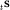 is then projected onto $I_{k}$ (see Figure 3.5) with

(16)

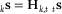

and is tracked with OpenCV's discriminative correlation filter tracker (CSRT) during the closed‐loop visual servoing (see Figures 2 and 3.6).^[^ ^34^ ^]^ The CSRT tracker has performed well in tracking targets on endoscopic images during ex vivo experiments with human placentas.^[^ ^14^ ^]^

### 2.4 In Vitro Evaluation of Exploration Motions

**Figure 4:**
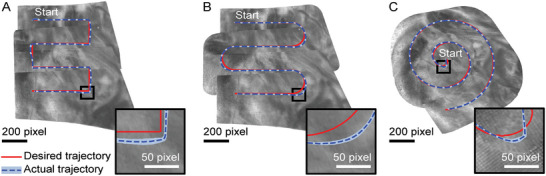
In vitro evaluation of the exploration phase. (A–C) show the desired trajectory (red) and the mean and standard deviation of ten runs (dashed blue) in the mosaic frame. The zoom shows the area with the lowest accuracy for each trajectory. The mosaic generated in the first run is shown in grayscale in the background.

The performance of the exploration of the environment while following a predefined trajectory in the mosaic frame was evaluated by navigating over an image of the human placenta used in the ex vivo experiments. We compared three patterns that are commonly used to generate mosaics,^[^ ^19^, ^26^, ^27^ ^]^ namely two types of raster patterns and a spiral trajectory (see red desired trajectories in **Figure** **4**). The trajectories were followed at the desired speed of 10 pixels.s^‐1^ in the mosaic frame. Each pattern was repeated ten times. The Image Jacobian J was calibrated before each run to account for internal stresses in the endoscope. The initial magnetic field vector was determined prior to the first run of each pattern and kept constant per pattern.

The desired and measured trajectories are resampled over time to have the same amount of samples N for all runs. For each sample n of a given pattern in run j, we define the error $e_{n,j}$ as

(17)

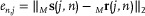

which corresponds to the Euclidean distance between the desired point along the trajectory 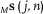 and the projected center of the endoscopic image $I_{n}$ in the mosaic 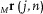. For each sample, the accuracy and repeatability of the tracking over the runs are evaluated as the mean and standard deviation of the error for this sample over the runs:

(18) $${\bar{e}}_{n}=\frac{1}{N_{r}}\sum_{j}e_{n,j}$$

(19) $$\sigma_{n}=\sqrt{\frac{1}{N_{r}}\sum_{j}{\left(e_{n,j}-{\bar{e}}_{n}\right)}^{2}}$$

with $N_{r}=10$ the number of runs. The accuracy and repeatability for each pattern are then calculated as the mean of ${\bar{e}}_{n}$ and $\sigma_{n}$ over the samples, which we denote $e_{P}$ and $\sigma_{P}$, respectively.

The accuracy and repeatability of the exploration motions for each pattern are shown in **Table** **1**. The mean accuracy $e_{P}$ ranged from 14 pixels for the spiral pattern to 17 pixels for the other patterns. The repeatability was high for all motions, with $\sigma_{P}$ ranging from 2.2 to 2.3 pixels. Figure 4 shows the comparison between the desired and actual trajectory over the mosaic. The zoomed‐in image shows the area with the lowest accuracy (i.e., the highest ${\bar{e}}_{n}$) and illustrates high repeatability at these locations. The background images are the grayscale version of the mosaics generated from the first runs. If a higher accuracy is required, J could either be re‐calibrated when the error gets too large, or J could be updated in real time.^[^ ^14^ ^]^

**Table 1:** Performance statistics of the exploration phase.

	Trajectory	Accuracy *e*_*P*_ [pixel]	Repeatability σ_*P*_ [pixel]	Mean time [s]	Number of samples *N*
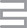	Raster	17±7.5	2.3±0.91	239	2391
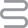	Raster rounded	17±7.7	2.2±1.0	274	2735
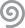	Spiral	14±5.0	2.3±0.87	299	2993

### 2.5 In Vitro Evaluation of the Navigation Phase

**Figure 5:**
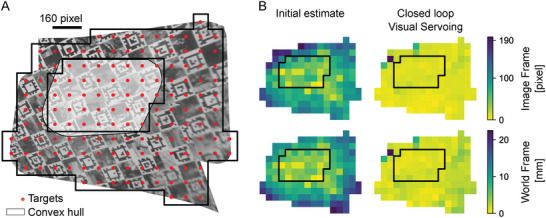
In vitro evaluation of the navigation phase. A) Mosaic and targets used for the evaluation. B) Error to reach targets in the mosaic. Each box corresponds to a target. The black border indicates the convex hull. The error was evaluated in the image frame (top row) and in the world frame (bottom row). The errors after the initial estimate of the magnetic field (left column) and after closed loop visual servoing (right column) are shown.

The accuracy of the navigation to targets in the mosaics was evaluated in an automated manner in vitro by navigating the endoscope over a plate with visual fiducials (AprilTags) to obtain ground truth for all target locations.^[^ ^35^, ^36^ ^]^ The fiducials were placed like a checkerboard, where each black square corresponded to a fiducial, and the white squares were filled with small feature‐rich images to ensure smooth mosaicking of the endoscopic images (see **Figure** **5**A). First, the mosaic was generated by following a raster trajectory with rounded edges (like the one in Figure 4B). The bending angle of the endoscope ranged from 0 to 25° during this motion and the 3D trajectory of the endoscope's tip is shown in Figure S1 (Supporting Information). The endoscope was then steered to 149 targets, which were generated over the mosaic with a spacing of 80 pixels (see Figure 5A). The order of the targets was chosen in a random manner. A target was considered to be reached if the tracked target was within a radius of three pixels around the image center (see Video S1, Supporting Information).

The results are depicted in Figure 5B. The error was calculated in the image frame and in the world frame (origin at the top left corner of the plate). It was evaluated for each target after the initial estimation of the magnetic field and after closed‐loop visual servoing. The error was defined in the image frame as the Euclidean distance between the center of the image and the target, and in the world frame as the Euclidean distance between the projection of the target and the projected image center. The results clearly illustrate that the inaccuracies of initial estimates can be significantly reduced by adding loop‐closure through visual servoing. The median error and median absolute deviation in the image frame was 103±33 pixels for the magnetic field estimation and 8.16±1.5 pixels after visual servoing (see **Table** **2** for the results in the world frame). The error of the magnetic field estimation is larger for the targets outside the convex hull H calculated from the projected image centers of the endoscopic images forming the mosaic and more uniformly distributed after visual servoing. This is because the magnetic field is only interpolated at locations inside H. If the target lies outside H, the target is first projected onto the closest point on H and the magnetic field is estimated at this location to avoid extrapolation (see Figure 3.2).

**Table 2:** Median and Median Absolute Deviation during Navigation in Mosaic.

	Initial estimate	Closed loop visual servoing
Image Frame [pixel]	100±33	8.2±1.5
World Frame [mm]	9.00±3.7	0.66±0.18

### 2.6 Ex vivo Demonstration

To simulate the application of our proposed navigation strategies for the treatment of TTTS, the endoscope was steered over an ex vivo human placenta underwater. The environment was first explored following a raster trajectory with rounded edges to generate a mosaic (see Video S2, Supporting Information). The endoscope was then steered to points of interest where the vessels could potentially be ablated.

**Figure 6:**
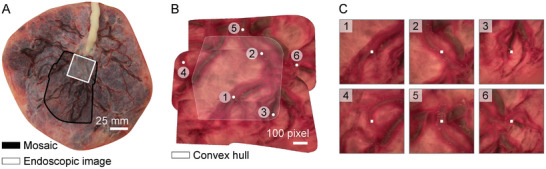
Ex vivo demonstration of the navigation phase. A) Human placenta used for the ex vivo demonstration. In black, the area explored by the mosaic, and in white the area of an endoscopic image is illustrated. B) Mosaic with 6 targets selected during the experiment (white disks). The light gray area is the convex hull calculated from the projected centers of the images forming the mosaic. Right: images 1–6 show the endoscopic image after the target was considered to be reached by the center of the endoscopic image (white squares).

**Figure** **6**A shows a mosaic generated from the exploration phase. The mosaic was then used as a user interface, and different target locations were manually selected (white disks in Figure 6B). Targets 1–3 were chosen inside the convex hull H generated from the centres of the projected images forming the mosaic, and targets 4–6 were selected outside H. The endoscope autonomously navigated to the target locations so that the target appeared at the center of the endoscopic image. A target was considered to be reached if the error between the tracked target and the endoscopic image center were below ten pixels. Figure 6 shows the endoscopic images after the target were reached and the center of each image (white squares). All targets were successfully reached. A sequence of the generation of the mosaic, as well as the navigation to different targets is shown in the Video S3 (Supporting Information). If a target was not reached with a sufficient accuracy, the user could simply switch to the visual servoing approach proposed in our previous work, where the endoscope is navigated to targets visible in the endoscopic images.^[^ ^14^ ^]^

## 3 Conclusion and Perspectives

In this work, we demonstrated the first automated navigation strategy to explore and navigate in large, hollow anatomical structures with RMN. We proposed two automated navigation strategies for RMN relying on endoscopic mosaics. During the exploration phase, the endoscope autonomously explores its environment with our first navigation strategy while continuously stitching its images into a mosaic. This provides to the surgeon with a map of the environment, facilitates surgical planning, tool localization, and characterization of areas of interest.^[^ ^15^, ^16^, ^17^, ^18^, ^19^, ^20^, ^21^, ^26^, ^27^, ^37^ ^]^ During the navigation phase, the generated mosaic can then be used as an interactive map where the user can select target positions to which the endoscope is automatically guided with our second proposed strategy. In this way, the navigation to targets is efficient and intuitive. We demonstrated their performance in feature‐rich and challenging low‐texture environments and navigated in air and underwater. In the context of TTTS, we showed their potential for FLC by navigating underwater over an ex vivo placenta. We were able to generate seamless mosaics and successfully reached all targeted locations on the placental vessels.

The navigation was performed using a constant insertion depth of the endoscope. A method to estimate the distance to the surface and navigate at a constant distance can be implemented. Distance detection with endoscopes is an active area of research and several methods ranging from additional sensors to vision‐based strategies have been proposed.^[^ ^38^, ^39^ ^]^

Enhanced methods to map surgical environments from endoscopic images are needed. Adding loop‐closure greatly improves the quality of the mosaics. Re‐localization of endoscopic images provides extra information to the user and facilitates transition to closed‐loop visual servoing in the navigation phase. The target position can be directly projected from the mosaic to the current endoscopic image eliminating the need to record the images forming the mosaic. Finally, the size of our mosaics was limited by large warping of the endoscopic images at the borders of the mosaic. Thus, 3D reconstructions instead of mosaics could enable even larger maps of the environment.

The navigation strategies, we proposed can be used independently and beyond the context of TTTS treatment. During gastroscopies or cystoscopies, the inner walls of the stomach or bladder are manually scanned with endoscopes searching for lesions such as ulcers and cancer or assessing their evaluation.^[^ ^16^, ^17^ ^]^ Mosaicking or 3D reconstructions of these organs allow on one hand the visualization of entire lesions with a high image resolution and on the other hand facilitate their localization within the organs, which is important to determine future treatment.^[^ ^16^, ^17^ ^]^ This work opens two new opportunities in this context: 1) the automation of the scanning of those organs while generating image mosaics or 3D reconstructions and 2) the navigation within these mosaics to reliably reach areas of interests for closer inspection or intervention. Our navigation strategies could even be used in combination with different imaging modalities such as confocal endomicroscopy, micro optical coherence tomography or ultrasound.

While RMN is a promising technology for minimally invasive procedures in general, it does exhibit some limitations and implementation challenges when used to steer soft surgical tools. These are usually more difficult to control, localize, and model than their rigid counter parts.^[^ ^4^, ^11^ ^]^ These limitations are alleviated by the fact that our approach is model‐free, and can fully adapt to various visual environments. This avoids the need to model realistic clinical environments, which can be a major challenge for their control.^[^ ^4^ ^]^

## 4 Experimental Section

### Experimental Setup

The performance of the navigation methods was evaluated for a flexible, magnetic endoscope and the Navion eMNS. In vitro experiments were conducted to quantify the performance of the proposed methods for the exploration and navigation phase in simplified visual environments. By navigating over an ex vivo human placenta underwater, the feasibility of the proposed methods was demonstrated in a low‐texture environment and showed their potential for FLC to treat TTTS. The Human placentas were donated with informed written consent and the approval of the Ethical Committee of the District of Zürich (BASEC‐Nr.: 2023‐00110).

### Setup

The field orientation at the beginning of each experiment was chosen such that the endoscope pointed downward facing the visual environment. The endoscope was guided within a trocar that was fixed in the vertical direction at 18 cm in front of the Navion. The distance from the surface to the tip of the endoscope was 3.6 mm for the in vitro experiment with the visual fiducials and 7 mm for the others. A field strength of 17 mT was used to evaluate the trajectory during the exploration motions and 20 mT for the remaining experiments.

### Endoscope

The endoscope was designed for FLC and contains a CMOS camera at the tip (resolution 400 × 400, OVM6946 by omnivision), illumination, and a channel for a laser fiber. Its diameter of 3.2 mm and ensures that it fits through the 10 Fr trocar used for FLC. An image of the endoscope is shown in Figure S2 (Supporting Information).

### Visual Environments

A plate with a grid of visual fiducials on it (AprilTags, tag family 41h12, size of 4.5 mm^[^ ^35^, ^36^ ^]^). This visual environment was chosen so that the pose of the camera with respect to the environment can be estimated for each endoscopic image and, thus, enables the calculation of ground truth errors for the navigation phase.

A photo of a human placenta used in the ex vivo experiments to provide a realistic, low‐texture visual environment for the endoscope. It was printed such that the photo was the same size as the real placenta. For repeatability experiments, the picture of the placenta was chosen instead of the real placenta, because the placenta was constantly leaking blood and its membranes moved slightly in the water.

The ex vivo experiment with a human placenta was performed to evaluate the potential of the proposed methods for FLC. The placenta and the endoscope were placed underwater to simulate realistic conditions.

## Conflict of Interest

The authors declare no conflict of interest.

## Supporting information

Supporting Information

Supplemental Video 1

Supplemental Video 2

Supplemental Video 3

## Data Availability

The data that support the findings of this study are available from the corresponding author upon reasonable request.

## References

[1] P. E. Dupont , B. J. Nelson , M. Goldfarb , B. Hannaford , A. Menciassi , M. K. O'Malley , N. Simaan , P. Valdastri , G. Z. Yang , Sci. Rob. 2021, 6, 60. 10.1126/scirobotics.abi8017 PMC8890492 34757801

[2] Y. Kim , E. Genevriere , P. Harker , J. Choe , M. Balicki , R. W. Regenhardt , J. E. Vranic , A. A. Dmytriw , A. B. Patel , X. Zhao , Sci. Rob. 2022, 7, 65. 10.1126/scirobotics.abg9907 PMC9254892 35417201

[3] G. Bassil , S. M. Markowitz , C. F. Liu , G. Thomas , J. E. Ip , B. B. Lerman , J. W. Cheung , J. Cardiovasc. Electrophysiol. 2020, 31, 739. 32022316 10.1111/jce.14380

[4] J. Edelmann , A. J. Petruska , B. J. Nelson , J. Med. Robot. Res. 2018, 03, 1850002.

[5] J. J. Abbott , E. Diller , A. J. Petruska , Ann. Rev. Control, Robot., Auton. Syst. 2020, 3, 57.

[6] D. V. Kladko , V. V. Vinogradov , Smart Mater. Med. 2023, 5, 24.

[7] A. Zemmar , A. M. Lozano , B. J. Nelson , Nat. Mach. Intell. 2020, 2, 566.

[8] J. W. Martin , B. Scaglioni , J. C. Norton , V. Subramanian , A. Arezzo , K. L. Obstein , P. Valdastri , Nat. Mach. Intell. 2020, 2, 595. 33089071 10.1038/s42256-020-00231-9 PMC7571595

[9] M. N. Faddis , J. Chen , J. Osborn , M. Talcott , M. E. Cain , B. D. Lindsay , J. Am. Coll. Cardiol. 2003, 42, 1952. 14662258 10.1016/j.jacc.2003.07.023

[10] C. Fischer , Q. Boehler , B. J. Nelson , IEEE Robot. Autom. Lett. 2022, 7, 7217.

[11] R. Dreyfus , Q. Boehler , B. J. Nelson , IEEE Robot. Autom. Lett. 2022, 7, 8370.

[12] A. Hong , A. J. Petruska , A. Zemmar , B. J. Nelson , IEEE Trans. Biomed. Eng. 2021, 68, 616. 32746060 10.1109/TBME.2020.3009693

[13] S. Gervasoni , J. Lussi , S. Viviani , Q. Boehler , N. Ochsenbein , U. Moehrlen , B. J. Nelson , IEEE Trans. Med. Robot. Bion. 2022, 4, 85.

[14] J. Lussi , S. Gervasoni , M. Mattille , R. Dreyfus , Q. Boehler , M. Reinehr , N. Ochsenbein , B. J. Nelson , U. Moehrlen , Adv. Intell. Syst. 2022, 4, 2200182.

[15] K. L. Lurie , R. Angst , D. V. Zlatev , J. C. Liao , A. K. Ellerbee Bowden , D. Ai , J. Yang , J. Fan , Y. Zhao , X. Song , J. Shen , L. Shao , Y. Wang , A. J. Das , T. A. Valdez , J. A. Vargas , P. Saksupapchon , P. Rachapudi , Z. Ge , J. C. Estrada , R. Raskar , e. Baek , J. S. Lim , W. J. Hyung , G. F. Riley , A. L. Potosky , J. D. Lubitz , L. G. Kessler , Biomed. Opt. Express 2017, 8, 2106. 28736658 10.1364/BOE.8.002106 PMC5516821

[16] T. B. Phan , D. H. Trinh , D. Wolf , C. Daul , Pattern Recognit. 2020, 105, 107391.

[17] A. R. Widya , Y. Monno , M. Okutomi , S. Suzuki , T. Gotoda , K. Miki , IEEE J. Transl. Eng. Health Med. 2021, 9, 1. 10.1109/JTEHM.2021.3062226 PMC8009143 33796417

[18] O. Alabi , S. Bano , F. Vasconcelos , A. L. David , J. Deprest , D. Stoyanov , Int. J. Comput. Assist. Radiol. Surg. 2022 2022, 17, 1125. 10.1007/s11548-022-02623-1 PMC9124660 35503395

[19] B. Rosa , M. S. Erden , T. Vercauteren , B. Herman , J. Szewczyk , G. Morel , IEEE Trans. Biomed. Eng. 2013, 60, 1041. 23192481 10.1109/TBME.2012.2228859

[20] S. Bano , F. Vasconcelos , A. L. David , J. Deprest , D. Stoyanov , Comput. Methods Biomech. Biomed. Eng.: Imaging Vis. 2023, 11, 1166.

[21] L. Li , E. Mazomenos , J. H. Chandler , K. L. Obstein , P. Valdastri , D. Stoyanov , F. Vasconcelos , Med. Image Anal. 2023, 84, 102709. 36549045 10.1016/j.media.2022.102709 PMC10636739

[22] S. Bano , A. Casella , F. Vasconcelos , A. Qayyum , A. Benzinou , M. Mazher , F. Meriaudeau , C. Lena , I. A. Cintorrino , G. R. De Paolis , J. Biagioli , D. Grechishnikova , J. Jiao , B. Bai , Y. Qiao , B. Bhattarai , R. R. Gaire , R. Subedi , E. Vazquez , S. Płotka , A. Lisowska , A. Sitek , G. Attilakos , R. Wimalasundera , A. L. David , D. Paladini , J. Deprest , E. De Momi , L. S. Mattos , S. Moccia , et al., Med. Image Anal. 2024, 92, 103066.38141453 10.1016/j.media.2023.103066

[23] C. Bamberg , K. Hecher , Best Pract. Res. Clin. Obstet. Gynaecol. 2019, 58, 55. 30850326 10.1016/j.bpobgyn.2018.12.011

[24] V. M. Pandya , J. Stirnemann , C. Colmant , Y. Ville , Y. Pan , D. D. Shi , Maternal‐Fetal Med. 2020, 2, 34.

[25] L. Van Der Veeken , I. Couck , J. Van Der Merwe , L. De Catte , R. Devlieger , J. Deprest , L. Lewi , Facts, Views Vis. ObGyn 2019, 11, 197. 32082525 PMC7020942

[26] L. Zhang , M. Ye , P. Giataganas , M. Hughes , G. Z. Yang , in 2017 IEEE International Conference on Robotics and Automation (ICRA) , Institute of Electrical and Electronics Engineers Inc., IEEE, Piscataway, NJ 2017, pp. 3587–3593.

[27] X. Xu , R. Tang , L. Gong , B. Chen , S. Zuo , IEEE Robotics and Automation Letters 2021, 6, 5728.

[28] G. Fagogenis , M. Mencattelli , Z. Machaidze , B. Rosa , K. Price , F. Wu , V. Weixler , M. Saeed , J. E. Mayer , P. E. Dupont , Sci. Rob. 2019, 4, eaaw1977. 10.1126/scirobotics.aaw1977 PMC6693882 31414071

[29] J. Han , J. Davids , H. Ashrafian , A. Darzi , D. S. Elson , M. Sodergren , Int. J. Med. Robot. Comput. Assist. Surg. 2022, 18, e2358. 10.1002/rcs.2358 34953033

[30] J. Sikorski , A. Denasi , G. Bucchi , S. Scheggi , S. Misra , IEEE/ASME Trans. Mechatron. 2019, 24, 505.

[31] A. J. Petruska , J. Edelmann , B. J. Nelson , IEEE Trans. Magn. 2017, 53, 1.

[32] S. Bano , F. Vasconcelos , L. M. Shepherd , E. Vander Poorten , T. Vercauteren , S. Ourselin , A. L. David , J. Deprest , D. Stoyanov , Medical Image Computing and Computer Assisted Intervention – MICCAI 2020, Springer, Berlin, Heidelberg 2020, pp. 763–773.

[33] S. Bano , F. Vasconcelos , A. L. David , J. Deprest , D. Stoyanov , in Hamlyn Symposium on Medical Robotics, The Hamlyn Centre Faculty of Engineering Imperial College London, London, UK 2022.

[34] A. Lukežič , T. Vojíř , L. E. Zajc , J. Matas , M. Kristan , Proceedings ‐ 30th IEEE Conference on Computer Vision and Pattern Recognition, CVPR , IEEE, Piscataway, NJ 2017, pp. 4847–4856.

[35] E. Olson , 2011 IEEE International Conference on Robotics and Automation , IEEE, Piscataway, NJ 2011, pp. 3400–3407.

[36] J. Wang , E. Olson , 2016 IEEE/RSJ International Conference on Intelligent Robots and Systems (IROS) , IEEE, Piscataway, NJ 2016, pp. 4193–4198.

[37] J. Liu , B. Wang , W. Hu , P. Sun , J. Li , H. Duan , J. Si , IEEE Trans. Biomed. Eng. 2015, 62, 2296. 25910000 10.1109/TBME.2015.2424438

[38] C. Gruijthuijsen , R. Colchester , A. Devreker , A. Javaux , E. Maneas , S. Noimark , W. Xia , D. Stoyanov , D. Reynaerts , J. Deprest , S. Ourselin , A. Desjardins , T. Vercauteren , E. V. Poorten , J. Med. Robot. Res. 2018, 3, 3n41841001. 10.1142/S2424905X18410015 PMC6390942 30820482

[39] Z. Fu , Z. Jin , C. Zhang , Z. He , Z. Zha , C. Hu , T. Gan , Q. Yan , P. Wang , X. Ye , IEEE Access 2021, 9, 41144.

