# Supplementary material for: Autonomous Magnetic Navigation in Endoscopic Image Mosaics
Source: Adv Sci (Weinh). 2024 Mar 14;11(19):2400980. doi: 10.1002/advs.202400980 (PMC11109657; doi:10.1002/advs.202400980)
Supplement: Supplementary file 1 — Supporting Information [file ADVS-11-2400980-s001.pdf]

ADVANCED SCIENCE
Open Access

Supporting Information

for *Adv. Sci.*, DOI 10.1002/advs.202400980

Autonomous Magnetic Navigation in Endoscopic Image Mosaics

Michelle Mattille, Quentin Boehler*, Jonas Lussi, Nicole Ochsenbein, Ueli Moehrlen and Bradley J. Nelson*

Supporting Information
Autonomous Magnetic Navigation in Endoscopic Image Mosaics

Michelle Mattille Quentin Boehler* Jonas Lussi Nicole Ochsenbein Ueli Moehrlen Bradley J. Nelson*

1 Supporting Information

Supplementary Figures:

Figure S1. Trajectories of the endoscopic tip with respect to the initial pose during *in vitro* exploration

Figure S2. Image of the magnetic endoscope

Supplementary Videos:

Video S1 (.mp4 format). Automated evaluation procedure

Video S2 (.mp4 format). Automated generation of mosaic

Video S3 (.mp4 format). *Ex vivo* demonstration of the workflow

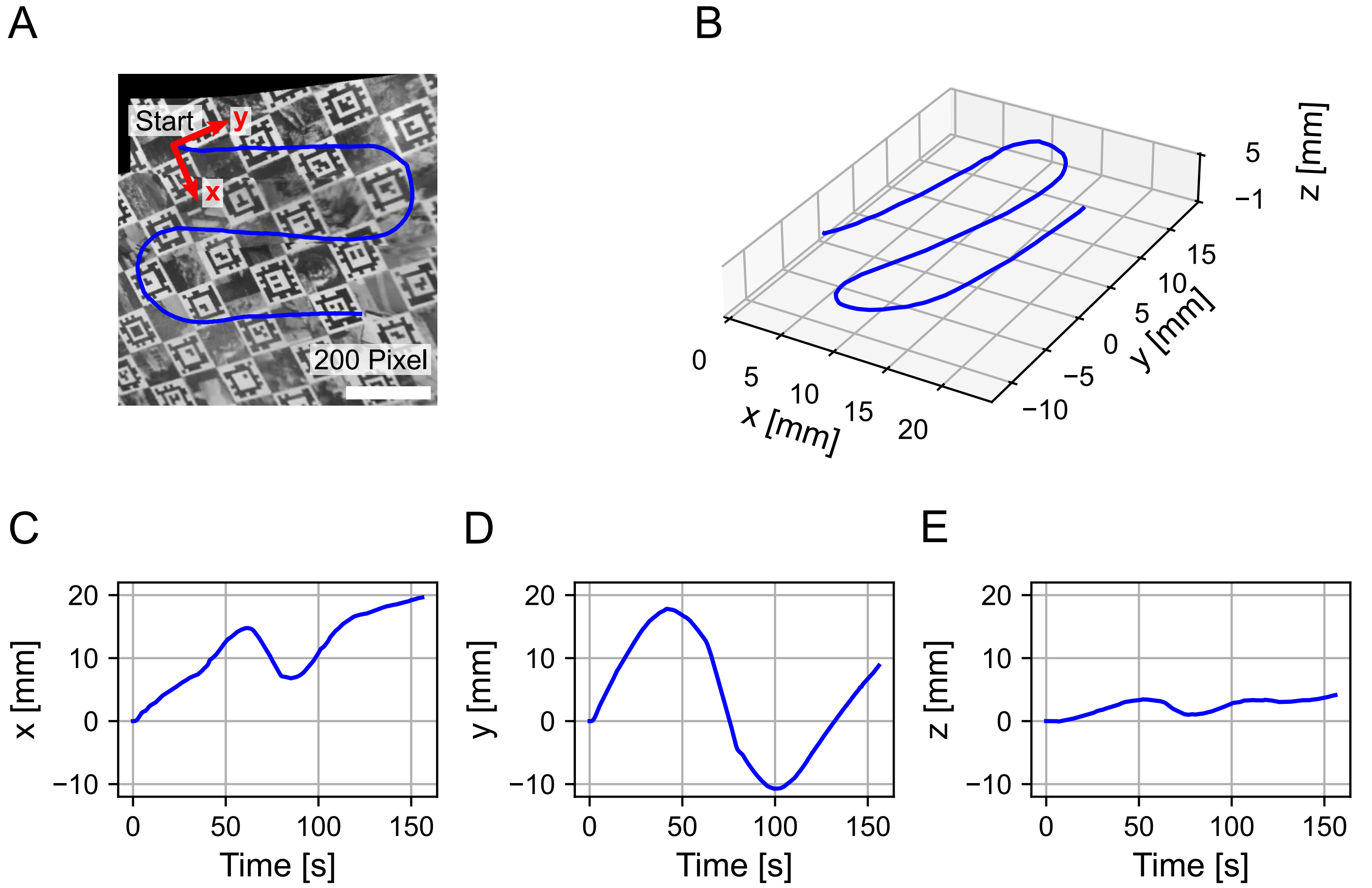

Figure S1: Trajectories of the endoscopic tip with respect to the initial pose during in vitro exploration. A) Trajectory of the endoscopic image centers in the mosaic frame in blue and the generated mosaic in grayscale in the background. In red the local frame used in B-E is depicted. Its axis point along the main directions of the visual fiducials on the plate and its origin is the initial position of the endoscope during in vitro exploration. B) 3D Trajectory of the endoscopic tip with respect to the initial pose in the local frame. C-E) Projections of the 3D trajectory along the different axis of the local frame.

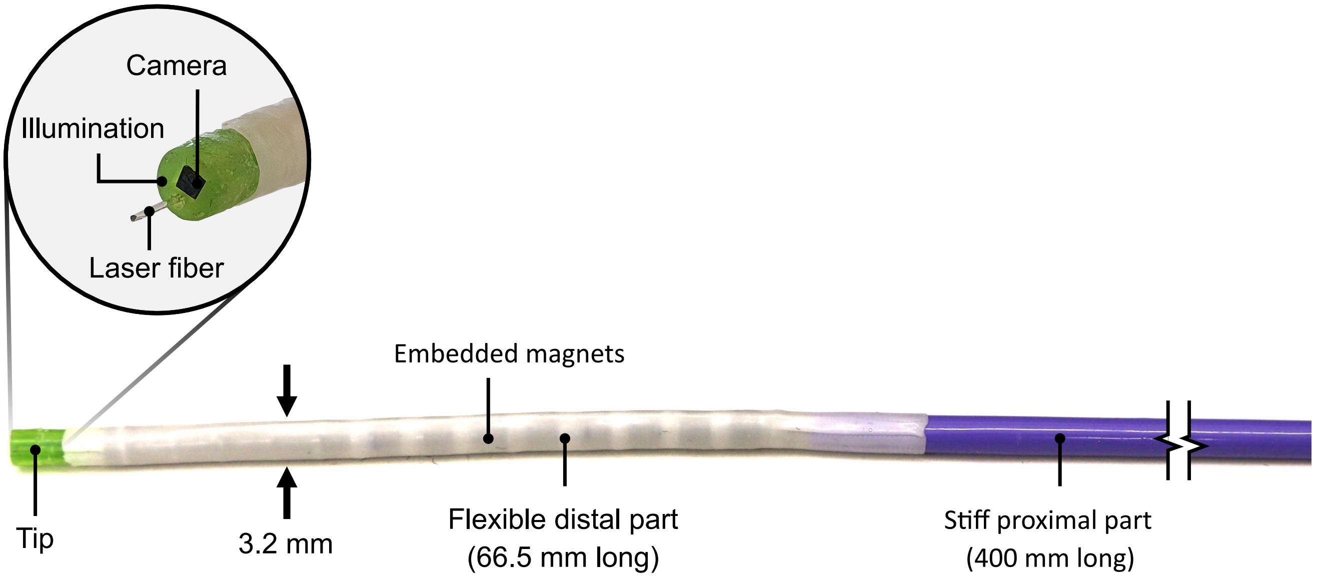

Figure S2: Image of the magnetic endoscope. It contains a working channel for a laser fiber as well as a camera and optical fibers for illumination. Its steerable, distal part is flexible and contains embedded magnets whereas the stiff proximal part enhances stability of the device in the changing magnetic fields.
